# Predicting the Linguistic Accessibility of Chinese Health Translations: Machine Learning Algorithm Development

**DOI:** 10.2196/30588

**Published:** 2021-10-07

**Authors:** Meng Ji, Pierrette Bouillon

**Affiliations:** 1 School of Languages and Cultures University of Sydney Sydney Australia; 2 University of Geneva Geneva Switzerland

**Keywords:** machine learning, health translation, Chinese health resources

## Abstract

**Background:**

Linguistic accessibility has an important impact on the reception and utilization of translated health resources among multicultural and multilingual populations. Linguistic understandability of health translation has been understudied.

**Objective:**

Our study aimed to develop novel machine learning models for the study of the linguistic accessibility of health translations comparing Chinese translations of the World Health Organization health materials with original Chinese health resources developed by the Chinese health authorities.

**Methods:**

Using natural language processing tools for the assessment of the readability of Chinese materials, we explored and compared the readability of Chinese health translations from the World Health Organization with original Chinese materials from the China Center for Disease Control and Prevention.

**Results:**

A pairwise adjusted *t* test showed that the following 3 new machine learning models achieved statistically significant improvement over the baseline logistic regression in terms of area under the curve: C5.0 decision tree (95% CI –0.249 to –0.152; *P*<0.001), random forest (95% CI 0.139-0.239; *P*<0.001) and extreme gradient boosting tree (95% CI 0.099-0.193; *P*<0.001). There was, however, no significant difference between C5.0 decision tree and random forest (*P*=0.513). The extreme gradient boosting tree was the best model, achieving statistically significant improvement over the C5.0 model (*P*=0.003) and the random forest model (*P*=0.006) at an adjusted Bonferroni *P* value at 0.008.

**Conclusions:**

The development of machine learning algorithms significantly improved the accuracy and reliability of current approaches to the evaluation of the linguistic accessibility of Chinese health information, especially Chinese health translations in relation to original health resources. Although the new algorithms developed were based on Chinese health resources, they can be adapted for other languages to advance current research in accessible health translation, communication, and promotion.

## Introduction

Translation serves as an important educational tool for health education and health promotion among multilingual and multicultural populations [1-3]. Health literacy research shows that improving the linguistic accessibility and understandability of health translations can have an important impact on the uptake of health recommendations by medical professionals and health authorities [4]. Current approaches to multicultural health resource evaluation are chiefly qualitative and use clinically developed guidelines or the judgement of health professionals [5,6]. There are several limitations to these approaches. First, there is the potential inconsistency of evaluation among medical professionals. Second, generalized, principled evaluation of health agencies tends to have low adaptability or flexibility, but users of health translations represent vulnerable populations who vary in their language, culture, education backgrounds, cognitive abilities, and health literacy [7]. Third, evaluation by experts requires longer timeframes, particularly with large quantities of translation resources. In situations of health emergencies which require rapid, more regular communication of information of health risk prevention or management, this can be technically challenging. Finally, logistically, the expert evaluation of health translations in minority languages can be costly [8,9] or is simply not available when there is a lack of suitably qualified medical professionals with adequate knowledge and understanding of minority languages as the target languages of health translations.

In medical and health research, an established approach to the quantitative evaluation of health education resources is the use of readability tools. Some widely used comparable readability tools include Flesch reading ease score, the Gunning fog index, Flesch-Kincaid grade level readability, the Coleman-Liau index, the SMOG index, the automated readability index, and the Linsear Write formula [10-14]. The mathematical design and the functions of these readability tools primarily focus on 3 large dimensions of the linguistic accessibility of health resources for the public: morphological complexity (average number of syllables per word, average characters per word, and average number of letters per word), syntactic complexity (average sentence length and average number of words in sentences), and semantic complexity (percentage of hard words). These readability tools offer fast, convenient measurements of original health resources and provide instant evaluation of the suitability of a new health text for readers of a certain education level. They can also be applied in the study of translated resources in English or European languages.

The limitation of these readability diagnostic tools is also known. First, the measurement of linguistic or textual complexity at the morphological level is based on the calculation of syllables or letters which can hardly be applied in languages that use different alphabets or symbols. For example, East Asian languages like Chinese, Japanese Kanji, and Korean use strokes instead of letters or syllables. Even within the same language, written language varieties; for example, the traditional or simplified versions of Chinese, or the hiragana, katakana, and romanji of Japanese can compound the measurement of linguistic complexity significantly. Second, existing medical readability formulae tend to exploit the orthographical or sentential structures of texts. The design of these readability formulae assumes that linguistic readability can be explained or controlled by reducing the length of individual words, sentences, or the frequency of occurrence of specialized terminology. This assumption has simplified the complexity of the cognitive mechanism that underlies the reading and understanding of specialist health information [15,16]. This includes notably textual logic or coherence devices, such as pronouns, personal pronouns, or conjunctions. In corpus translation studies, the enhanced use of these functional linguistic devices is known as translational features or translationese [17-20]. However, whether these functional categories in translationese can be deployed systematically in professional health translations to increase the readability of translated health resources remains underexplored.

Our study developed new techniques to improve current approaches to Chinese health translation readability evaluation. First, we increased the dimensions of quantitative analyses by incorporating functional categories including pronouns, personal pronouns, conjunctions, and negative conjunctions to the existing measurements of morphological and sentential structural complexity. Second, we adapted the measurements of morphological complexity of European languages to character-based Asian languages. This included adding new morphological measurements of Chinese 2-character words, Chinese 3-character words, average strokes per character, high-stroke characters, low-stroke characters, and middle-stroke characters. Chinese 2- and 3-character words represent the common Chinese vocabulary; meanwhile, 4-character words are more associated with idioms or idiomatic expressions, and words of 4 or more characters are likely to be either specialized terminology, proper nouns, or translated expressions. The morphological complexity of Chinese characters is measured by the number of strokes in each character. High-stroke characters are comparable to polysyllabic words in English or European languages.

In our study, a third adaption to the existing medical readability tools involved the expansion of semantic categories. Among 7 widely used medical calculators, only the Linsear Write formula and the Gunning fog index incorporate “hard,” “difficult,” and “easy” words in the calculation of the linguistic readability of health translations. The addition of words of varying cognitive difficulty represents an advance from the quantification of health information readability based on word or sentence length, such as is done in the Flesch reading ease score, Flesch-Kincaid grade level readability, Coleman-Liau index, SMOG index, and automated readability index. However, the addition of easy versus hard words in Linsear Write formula and Gunning fog has an inherent methodological limitation, notably the lack of a clear, consistent definition of easy or hard words. The interpretation of lexical difficulty is open to the understanding of the users of these readability tools, thus causing inconsistency in the evaluation results among users of the same tool or between different readability tools. In this study, this inherent variability of the Linsear Write formula and Gunning fog was controlled by 6 clearly defined, quantifiable linguistic features based on cognitive and corpus linguistic research on their relevance for the semantic complexity of texts.

Table 1 lists these semantic categories: type and token ratio, density of content words, difficulty words, ratios of noun phrases, normalized frequency of noun phrases, and sentences with complex semantic categories (ie, polysemes). Here, the definition of difficult words is based on the extraction of the 3000 most common Chinese character words in the Academia Sinica Balanced Corpus of Modern Chinese developed by Academia Sinica. Character words which are not listed in the top 3000 words are retrieved as difficult Chinese words. Although the current threshold level of easy versus difficult words based on the first 3000 words in the balanced Chinese corpus is subjective, this corpus approach provided a more transparent and consistent reference point for different users of the new readability system (Table 1). The use of standardized semantic categories instead of absolute values as in the Linsear Write formula and Gunning fog can help reduce the impact of the length of the texts on the readability evaluation results. This is particularly useful for the evaluation of the Chinese health translations collected in the World Health Organization (WHO) website, which are of varying lengths based on the health topics and genres. The standardized semantic categories are type-token ration (TTR; proportion of different words within the total words of a text), density of content words (the proportion of content words within the total words of a text), ratios of noun phrases (proportion of noun phrases within the total words of a text), and normalized frequency of noun phrases (proportion of noun phrases per 10,000 words).

**Table 1:** New multidimensional framework of the linguistic readability of health translations in Chinese.

Category	Features
Morphology	Two-character words, three-character words, average strokes per character, high-stroke characters (above 21 strokes), low-stroke characters, middle-stroke characters (11-20 strokes)
Sentences structure	Average sentences per paragraph, average words per sentence, simple sentences
Semantics	Type-token ratio, content words, density of content words, difficult words (beyond the most common 3000 words), ratios of noun phrases, normalized frequency of noun phrases, sentences with complex semantic categories (polysemes)
Logic and coherence	Conjunctions, positive conjunctions, negative conjunctions, personal pronouns, pronouns, adverbs of negation

## Methods

### Data Collection

With an increasing number of health translations accessible on the internet and rapidly developing computational techniques, the development of cost-effective, robust algorithms for the computerized evaluation of the linguistic accessibility of health translations has become possible. This study explored machine learning techniques to effectively analyze and diagnose the linguistic accessibility of health translations by professional translators of the WHO. Two comparable corpora were constructed containing professional Chinese health translations developed by the WHO (350 full-length translations). The reference materials used to compare with Chinese health translations were original, public-oriented health educational resources published by China Center for Disease Control and Prevention (CCDC). These resources are regarded as authoritative health information widely disseminated by governmental organizations, industrial sectors, and the media. The use of original Chinese health education resources instead of human evaluation of the linguistic accessibility of health resources has both its methodological advantages and limitations. First, human evaluation is known to be susceptible to inconsistency unless there are clear, well-defined criteria of selecting human evaluators, such as age, gender, educational background, health literacy, and cognitive abilities. Although this could help limit the variability in the human evaluation, the evaluation results can be hardly representative of larger, more diverse populations. Another practical issue is the lack of well-established, national guidelines of the level of health educational resources for the public in China. This stands in contrast with English-speaking countries where health authorities provide clinical guidelines or recommendations for the suitable readability level of health resources to ensure access to health information by the greater population. Furthermore, few Chinese health resources have been assessed by international health website accreditation authorities (HON.net). This made it difficult to identify and collect health education resources in Chinese that could meet international health resource development guidelines or clinical and research-based recommendations. The use of CCDC resources was based on 2 considerations. The first was the authority of these resources in China, as the CCDC is the national disease prevention authority and the health educational materials by the CCDC have wide circulation within the country. Second, the quality control provided by the CDCC website content editor ensures the usability and understandability of the resources for the public in China. In this study, during the construction of the subcorpus of original Chinese health educational resources, native Chinese speakers were instructed to select and collect resources intended by the public, rather than technical materials written for medical health professionals, such as disease epistemology or clinical research. This was facilitated by the design of the CCDC website, which has designated sections for public health education resources to describe and explain complex or common diseases and symptoms. This data collection strategy has its limitation: there is a lack of national guidelines of health resource development, and thus the readability or accessibility of health content is not regulated or controlled by national or organizational standards. The content difficulty of these original Chinese health resources on the CCDC website may well be mixed. To overcome this issue, a large number of full-length original Chinese articles were randomly collected from the CCDC website to match the corpus of Chinese health translations of the WHO. Descriptive statistics of original and translated Chinese health texts are given in Multimedia Appendix 1.

### Statistical Analysis

Multimedia Appendix 1 shows the differences between the 2 comparable corpora of translated and original health resources covering diverse health topics. The Chinese translations were collected from the website of the WHO, and the original Chinese health resources were published on the website of the CCDC. The *P* values were derived from the Mann-Whitney test with SPSS version 20 (IBM Corporation). The results show that there were statistically significant differences between the translated and original Chinese resources in 20 of the total 22 linguistic and textual features studied. It was found that at the morphological level, there were more low-stroke characters in the original Chinese resources (mean 407.76), which was 1.4 times more than in the translated Chinese health resources (mean 285.06). However, there were also more middle- and high-stroke characters (around 1.6 times) in the original Chinese health resources than in the translated Chinese ones. As a result, the average stroke per character of the original Chinese (mean 7.86) was significantly higher than that of the translated Chinese materials (mean 7.71; *P*=.01). There were more 2- and 3-character words in the original Chinese health resources (mean 2-character words 160.73; mean 3-character words 13.77) than in translated ones (mean 2-character words 114.49; mean 3-character words 8.29). Most of the modern Chinese lexis is made of 2 or 3 characters, suggesting that the lexical familiarity of original Chinese resources could be higher than the translation materials. Second, in terms of information load, the average TTR of the original Chinese texts (mean 0.59) was significantly lower than that of the translated texts (mean 0.62; *P*<.001). Similarly, the average words per sentences of the original Chinese texts (mean 10.83) was significantly lower than that of the translated Chinese resources (mean: 11.9: *P*<.001).

By contrast, the average sentences per paragraph of the original Chinese texts was almost double that of the Chinese health translations (mean 3.32; *P*<.001). This suggests that the translated health materials were longer and contained more information than did the original Chinese health texts and that the original Chinese materials featured longer paragraphs with more sentences despite the average sentence lengths being shorter. Another interesting finding was that there were more single sentences in the translated Chinese materials (mean 0.46) than in the original Chinese health texts (mean 0.32). This may be explained by the more frequent use of logical and coherence words in the original Chinese health texts than in the WHO translations. For example, there was a statistically significant higher use of conjunctions in the original Chinese (mean 14) than in the Chinese health translations (mean 11.53; *P*=.01), there were more negative conjunctions in the original Chinese (mean 2.03) than in the translations (mean: 1.44, *P*=.03), and there were more pronouns and personal pronouns in the original Chinese (mean: 2.36 for pronouns; 1.12 for personal pronouns) than in the Chinese health translations (pronouns: mean 1.47, *P*=0.01; personal pronouns: mean 0.7, *P*=.04). At the semantic level, although the difference between the original and translated Chinese health resources was not significant in terms of the ratio of noun phrases, the normalized frequency of noun phrases was much higher in the original (mean 321.89) than in the translated Chinese health texts (mean 314.58; *P*=.04). It was useful to notice that the mean of sentences with complex semantic categories (polysemes) was statistically higher—almost double—in the original Chinese resources (mean 14.42) compared to the translated Chinese ones (mean 7.61; *P*<.001). Finally, the density of content words in the original Chinese (mean 0.83) was statistically higher than that of the translated health texts (mean 0.81).

The statistical comparison (using the nonparametric test for 2 independent samples: Mann-Whitney test) indicated a mixed feature of the linguistic accessibility of original and translated Chinese health resources. It suggested that at the morphological level, the original Chinese health texts are more complex than are translated health texts, as the average number of strokes in characters was higher in the original Chinese texts than in the translated Chinese ones. However, this issue was somewhat offset by the higher use of more familiar lexis in the original text compared to the translated health materials, as the mean of 2- and 3-character words was significantly higher in the original Chinese health texts than in the Chinese health translations. Next, at the semantic level, the statistical comparison showed that the original Chinese health texts were more complex than the Chinese health translations, as evidenced by the higher density of content words and the higher mean of sentences with polysemes. However, this issue occurred in conjunction with the higher information load of Chinese health translations than of the original health texts, as illustrated by the higher TTRs and higher average words per sentences of health translations.

In terms of the logical structure and coherence of sentences, although there were more simple sentences in the Chinese health translations and more compound sentences in the original Chinese health texts, the latter featured more coherence devices such as pronouns and personal pronouns to assist with the reading and understanding of the original Chinese health texts. This mixed outcome of the comparison between the 2 comparable corpora suggests that the assessment of the linguistic accessibility of health resources should be balanced and multidimensional to avoid any partial, biased assessment outcome. The proposed multidimensional corpus analysis contrasts with the use of medical readability tools which focus on the morphological and syntactic features of health texts, such as word length in letters or syllables or sentence lengths in words. The machine learning modeling results to be shown demonstrate that semantic, logical, and coherence features are also equally important for building effective evaluation models of the linguistic accessibility of Chinese health translations.

### Development of Machine Learning Models

Machine learning models are different from conventional statistical methods. First, machine learning does not require a normal distribution to fit the data to existing statistical models. Machine learning is essentially data driven and is free to learn any functions underlying the training data without any implicit assumptions. This is especially the case for tree-based machine learning algorithms, such as gradient boost trees, decision trees, and random forest. With conventional statistical methods, the values of the independent variables and dependent variables must be both present to model their relationships, whereas with machine learning, once the algorithm has been well tested and validated, it can be used to predict the outcome of the target variable based on the information collected from the predictor variables. That is, machine learning can be used to make high-precision predictions. This suits the purpose of this study, whose aim was to develop a cost-effective health translation accessibility prediction model which can predict whether a certain health translation differs significantly from the original Chinese health education resources developed for the public of native Chinese speakers, for example, the health education resources we collected carefully from the website of the CCDC as the national health authority in China.

Effective machine learning models can instantly detect any potential reading barriers caused by linguistic accessibility issues of health translations, allowing translation and international health agencies to revise translations before their release to the public. Linguistically more accessible health translations in turn can help achieve better outcomes of health education programs and campaigns among vulnerable populations with limited English proficiency and low education and health literacy levels. In this study, random forest and extreme gradient boosting tree (XGBoost tree) were used to solve a classification problem: that is, to predict whether a certain Chinese health text is more likely to be of the linguistic accessibility level of an original Chinese health text or of a Chinese health translation based on the modelling of the linguistic features as the predictor variables to be extracted from an unlabeled health text in Chinese.

To overcome model overfitting, 5-fold cross-validation on the whole data set was conducted. In each 5-fold cross-validation, the entire data set was divided into 5 portions of approximately equal numbers. Four folds of data were used as the training data to develop the machine learning models, and the remaining fold was used as the test data set to calculate the model performance metrics including area under the receiver operating characteristic curve (AUC), sensitivity, specificity, and accuracy. After the iterations, each fold was used as the test set exactly once. The 5-fold cross-validation can help detect overfitting machine learning models that have large differences in the performance metrics of the 5 test data sets.

In this study, hyperparameter tuning of XGBoost tree involved the following steps. The maximum tree depth for base learners (max_depth) controls the depth of the tree. The greater the depth is, the more complex is the model and the higher are chances of the model overfitting. There is no standard value for adepts. Larger data sets require deep trees to learn the rules from a complex data set. The value ranges between 0 and infinity. In the cross-validation process, we set max_depth to the default value of 6. The number of estimators or boosted trees was set to the default value of 100. The minimum sum of instance weight needed in a child node (min_child_weight) is another effective overfitting prevention method. It is calculated by second-order partial derivatives and ranges between 0 and infinity. The larger the value, the more conservative the algorithm is. This was set to the default value of 1 in this study. The maximum delta step (max_delta_step) specifies the maximum step size that a leaf node can take. It ranges between 0 and infinity, and increasing the positive value will make the update step more conservative. It was set to 1 in this study. The learning objective was set to binary logistic regression, as the target variable had 2 outcome categories: the original Chinese health education resources and the translated Chinese health resources. The hyperparameter subsample refers to the subsample ratio of the training instance. For example, setting the subsample to 0.5 means that the algorithm randomly collects half of the entire data set to build the tree model. This method can prevent overfitting. The value of the subsample was set to 0.5 from its typical range of 0.5 to 0.8. Eta refers to the machine learning rate at which the algorithm learns the latent patterns and structures in the training data set. Smaller eta leads to slower computation and thus prevents overfitting. Smaller etas can be compensated for by increasing the number of boosted trees or estimators. Typically, the value of eta lies between 0.01 and 0.3, and 0.1 was set as the default value in this study. The hyperparameter colsample_bytree controls the number of features or variables supplied to a tree model, with a typical value ranging between 0.5 to 0.9, and it was set to 0.5 in this study. Lastly, α and λ values which control L1 and L2 regularization, were set to 1 and 0, respectively, to prevent overfitting.

Similar to XGBoost tree, random forest is another powerful ensemble learning technique that outperforms single learning algorithms in machine learning model development. In random forest, decision trees are used as the base learner, and bootstrapping aggregation combines these decision trees together to achieve high prediction accuracy. The minimum number of samples and training data required to be at a leaf node (min_samples_leaf) was set to 3. The maximum depth was set to 6. The number of features to use for splitting was set to auto. In the model construction process, the ensemble learning methods selected to increase the prediction accuracy included bootstrapping, bagging, and extremely randomized trees. In the process of hyperparameter optimization, on each iteration, the algorithm chooses a difference combination of the features. The maximum number of iterations was set to 1000, and the maximum evaluations was set to 300.

## Results

In the evaluation of the performance of the new machine learning models developed using XGBoost, random forest, and C5.0 decision tree, logistic regression was used as the baseline, as logistic regression has been used widely in both conventional statistical methods and traditional machine learning modelling. Table 2 shows that the logistic regression model was statistically significant. Table 3 shows the entered selection of important predictor variables in the final logistic regression model. It was found that among the initial 22 predictor variables (Multimedia Appendix 1), 4 predictor variables were identified as large contributing variables to the logistic regression model. These were average sentences per paragraph, middle-stroke characters, difficult words, and conjunctions. When the original Chinese health resources were used as the reference category, the Exp (B) values showed that textual features, including as higher average sentences per paragraph, higher middle-stroke characters, and higher use of difficult words were important features associated with the original Chinese health resources. For example, the average sentence per paragraph (*P*<.001) had an Exp (B) value of 0.44. This means that with the increase of one unit in average sentence per paragraph, the odds of the text being a Chinese health translation over the odds of the text being an original Chinese health text were 44%. Similarly, middle-stroke characters (*P*=0.03) had an Exp (B) value of 0.97. This suggests that with other variables being the same and with the increase of 1 middle-stroke character, the odds of the text being a Chinese health translation over the odds of the text being an original Chinese health text were 97%. Difficult words (*P*=.04) as the predictor variable had an Exp (B) value of 0.976. This means that with the increase of one difficult word, the odds of the text being a Chinese health translation were 2.4% lower than the odds of the text being an original Chinese health text. This finding suggests that higher use of difficult words is an important feature of original Chinese health educational resources when compared to Chinese health translations. By contrast, the predictor variable of conjunctions (*P*=.02) had an Exp (B) of 1.122, which means that with the other variables being the same and with the increase of 1 conjunction, the odds of the text being a Chinese health translation were 12.2% higher than the odds of the text being an original Chinese health education text. This corpus statistical finding can be explained by the theoretical hypotheses of translation studies such as translationese: the increased use of linguistic devices like conjunctions enhanced the textual cohesion of translated materials.

**Table 2:** Variables in the equation for the original reference category.

Variables	B	SE	Wald	Sig.^a^ (*P* value)	Exp(B)	95% CI for Exp (B)	
						Lower bound	Upper bound
Intercept	4.311	2.432	3.142	.07			
Average sentences per paragraph	–0.821	0.149	30.503	<.001	0.44	0.329	0.589
Frequency of noun phrases per 10,000 words	–0.008	0.005	2.732	.10	0.992	0.983	1.001
Average words per sentences	0.249	0.162	2.367	.12	1.282	0.934	1.76
Middle-stroke characters	–0.031	0.014	4.518	.03	0.97	0.943	0.998
Content words	0.005	0.009	0.291	0.59	1.005	0.987	1.023
Difficult words	–0.025	0.012	4.084	.04	0.976	0.953	0.999
Two-character words	0.012	0.011	1.053	.31	1.012	0.989	1.035
Conjunctions	0.115	0.047	5.93	.02	1.122	1.023	1.232
Sentences with complex semantic categories	0.006	0.064	0.008	.93	1.006	0.888	1.14
Ratio of noun phrases	0.719	0.995	0.521	.47	2.052	0.292	14.435
Pronouns	–0.1	0.148	0.461	.50	0.905	0.677	1.209
Personal pronouns	–0.071	0.198	0.127	.72	0.932	0.632	1.375
Negative conjunctions	0.12	0.146	0.677	.41	1.127	0.847	1.5

^a^sig: significance.

Table 3 shows the comparison of the AUC of the 3 machine learning models in comparison with the traditional logistic regression model: C5.0 decision tree has an AUC of 0.969, which is followed by random forest with an AUC of 0.957 and XGBoost tree with an AUC of 0.914. To evaluate the statistical significance between these models in terms of AUC improvement, the pairwise corrected resampled test was conducted as shown in Table 4. To overcome multiple comparison, the significance level was adjusted to 0.008 using Bonferroni correction. The result showed that the 3 new machine learning models (ie, C5.0 decision tree, random forest, and XGBoost tree) significantly improved the performance of the prediction, as their AUCs were significantly higher than the AUCs of logistic regression. There was no significant difference between C5.0 decision tree and random forest, which were significantly more precise than was XGBoost tree.

**Table 3:** Mean area under the receiver operating characteristic curve.

Test result variable(s)	AUC^a^	SE	Asymptotic sig.^b^ (*P* value)	Asymptotic 95% CI	
				Lower Bound	Upper Bound
XGBoost tree^c^	0.914	0.019	<.001	0.878	0.951
Random forest	0.957	0.014	<.001	0.93	0.984
C5.0 decision tree	0.969	0.012	<.001	0.945	0.992
Logistic regression (baseline)	0.768	0.025	<.001	0.718	0.818

^a^AUC: area under the curve.

^b^sig: significance.

^c^XGboost tree: extreme gradient boosting tree.

**Table 4:** Paired-sample *t* test of the area under the receiver operating characteristic curve.

Test result pair(s)	Asymptotic		AUC^a^ difference	SE difference^b^	Asymptotic 95% CI	
	*z*	Sig. (2-tailed *P* value)^c,d^			Lower bound	Upper bound
XGBT^e^-RF^f^	–2.74	.006	–0.043	0.179	–0.073	–0.012
XGBT-LR^g^	6.118	<.001	0.146	0.209	0.099	0.193
XGBT-C5^h^	–2.967	.003	–0.054	0.175	–0.09	–0.018
RF-LR	7.405	<.001	0.189	0.197	0.139	0.239
RF-C5	–0.655	.51	–0.011	0.161	–0.046	0.023
LR-C5	–8.094	<.001	–0.201	0.193	–0.249	–0.152

^a^AUC: area under the curve.

^b^Under the nonparametric assumption.

^c^Null hypothesis: true area difference = 0.

^d^A *P* value <0.008 is statistically significant (using Bonferroni correction). *P* values were derived from the pair-wise corrected resampled *t* test.

^e^XGBT: extreme gradient boosting tree.

^f^RF: random forest.

^g^LR: logistic regression.

^h^C5: C5.0 decision tree.

## Discussion

Next, through successive permutation, we examined the impact of textual features as predictor variables on the change in percentage of AUC in the best-performing machine learning algorithms, the C5.0 decision tree, and the baseline algorithm of logistic regression. Table 5 shows that a number of textual features contributed to changes in the mean AUC of logistic regression (0.768), notably, average sentences per paragraph (4.8%), simple sentences (2.6%), normalized frequency of noun phrases per 10,000 words (2.4%), average strokes per character (1.6%), conjunctions (1.5%), content words (1.3%), low-stroke characters (1.2%), and sentences with complex semantic categories (1.1%). By contrast, C5.0 decision tree which had a statistically better AUC (0.914) and featured a different set of textual features as key contributors to changes of the algorithm’s AUC. These included density of content words (7.3%), average words per sentence (3.2%), simple sentences (2.9), negative conjunctions (2.1%), positive conjunctions (2%), personal pronouns (1.8%), average sentences per paragraph (1.1%), high-stroke characters (1.1%), and TTR (1.1%). Only 2 textual features were identified as important contributors to both algorithms (causing a 1% or more decrease in AUC): simple sentences and average sentences per paragraph. Both simple sentences and average sentences per paragraph are measurements of the syntactic complexity of sentences. Existing medical readability formulae attempt to capture these features using average sentence length or average number of words in sentences. Morphological complexity was measured with different natural language features in the 2 algorithms. Logistic regression measured morphological complexity using average strokes per character and low-stroke (under 10 strokes) characters. C5.0 decision tree measured morphological complexity using high-stroke (over 20 strokes) characters.

**Table 5:** Changes in the area under the receiver operator characteristic curve through successive permutation of features.

Feature	LR^a^	C5^b^
Sentences with complex semantic categories	0.011	0
Content words	0.013	0
Low-stroke characters	0.012	0
Adverbs of negation	0.01	0
Average strokes per character	0.016	0
Two-character words	0	0.001
Conjunctions	0.015	0.001
Ratios of noun phrases	0.009	0.001
Normalized frequency of noun phrases per 10,000 words	0.024	0.001
Three-character words	0	0.002
Difficult words	0.01	0.002
Middle-stroke characters	0.001	0.004
Pronouns	0.001	0.008
Average sentences per paragraph	0.048	0.011
Type-token ratios	0.005	0.011
High-stroke characters	0.01	0.011
Personal pronouns	0.01	0.018
Positive conjunctions	0.009	0.02
Negative conjunctions	0.007	0.021
Simple sentences	0.026	0.029
Average words per sentences	0.007	0.032
Density of content words	0.01	0.073

^a^LR: logistic regression.

^b^C5: C5.0 decision tree.

Both machine learning algorithms identified and explored additional linguistic dimensions which were not studied in exiting medical resource readability assessment formulae. The first was information load, which refers to the amount and complexity of information contained in the texts. Logistic regression measured information load using natural language features, such as normalized frequency of noun phrases per 10,000 words and content words. Both categories contributed more than 1% of the changes in the AUC of logistic regression. Noun phrases are phrases which contain nouns and function as nouns in a sentence, and they are used extensively in medical and scientific writing. Higher normalized frequencies of noun phrases can significantly increase the information load of scientific discourse. C5.0 decision tree by contrast measured the information load of translated and original Chinese health resources using the density of content words, which was the percentage of content words of both content and function words in the Chinese texts. Content words include part-of-speech categories, including nouns, adjectives, adverbs, and verbs, whereas functional words comprise auxiliary verbs, pronouns, articles, and prepositions. A higher density of content words was found as a statistically significant feature of original Chinese health resources (mean 0.83) when compared to translated Chinese health resources (mean 0.81). TTR is another widely used measure in corpus linguistics to measure lexical diversity or richness. A higher TTR indicates an increased variety of words, and this was a significant feature of Chinese health translations (mean 0.62) when compared to the original Chinese health resources (mean 0.59).

Another dimension of linguistic features which was identified by the 2 machine learning algorithms based on natural language features was textual coherence and logical structure, which are not included in existing medical readability formulae. Logistic regression measured textual structure by using conjunctions, whereas the C5.0 decision tree algorithm exploited natural language features, such as negative conjunctions, positive conjunctions, pronouns, and personal pronouns. The original Chinese health resources featured a higher use of all these linguistic classes to heighten the logical structure of the Chinese health texts, whereas the translated Chinese health resources exhibited a more conservative use of these functional lexical categories. This finding cannot be explained by the hypothesized translationese or universal translation pattern of lexical simplification or normalization which is achieved through an increased use of functional devices such as conjunctions and pronouns. Rather, this may be a product of the influence from the source language, as English scientific discourse tends to use more passive sentence structures, whereas pronouns and personal pronouns are more common in everyday Chinese texts that use more direct, positive sentences. These linguistic features are related to the cognitive and logical properties of health texts. Although these textual features have not been incorporated into medical readability formulae, they are highly relevant to widely used health education resource development guidelines, for example, the Patient Education Materials Assessment Tool, which is a systematic method developed by the US Department of Health and Human Services to evaluate the understandability and actionability of patient education materials. Positive conjunctions and adverbs of negation may impact the logical sequence of health information, which is specified in the Patient Education Materials Assessment Tool as a key criterion for assessing the linguistic understandability of health educational resources.

Linguistic accessibility has an important impact on the reception and utilization of translated health resources among multicultural and multilingual populations with a high proportion of immigrants. Linguistic understandability of health translation has been understudied. Automated predictive analyses of the linguistic accessibility of new health translations before their release to the public can significantly improve the cost-effectiveness and efficiency of bilingual, multilingual health education programs and the use of health translation resources by the public. This paper introduced machine learning techniques to the study of the linguistic accessibility of health translations by comparing Chinese translations of the WHO materials with the original Chinese health resources developed by the Chinese health authorities. Three new machine learning models (XGBoost tree, random forest, and C5.0 decision tree) were developed and compared in terms of their accuracy, AUC, sensitivity, and specificity with the traditional logistic regression modeling being used as the baseline. The selection of textual features was based on existing research on corpus-based translation studies, cognitive linguistics, health literacy, and public health education. A number of textual and linguistic features were selected, which included morphological features, such as 2- and 3-character words, average strokes per character, low-stroke characters, and middle-stroke or high-stroke characters; features of sentential structures, such as average sentences per paragraph, average words per sentences, sentences with complex semantic categories, and single sentences; semantic features, such as TTRs, content words, density of content words, difficult words, and normalized frequency of noun phrases; and textual logic and coherence features, such as conjunctions, negative conjunctions, personal pronouns, and pronouns. Five-fold cross-validation was conducted in the model development process to ensure the reliability and replicability of the new machine learning models.

In the evaluation of the performance of the machine learning models, cross-model comparison was conducted. To counteract the issue of multiple comparisons and the risk of erroneous inferences, the significance level of the paired model comparison was adjusted to 0.008 using Bonferroni correction. The pairwise corrected resampled tests showed that C5.0 decision tree, random forest, and XGBoost tree all outperformed logistic regression with statistically higher AUCs. The impact of linguistic features on the AUC of the best-performing model, C5.0 decision tree, and the baseline logistic regression model was analyzed through eliminating 1 textual feature at a time from the whole set of textual features built within each algorithm. It was found that, like most existing linguistic readability evaluation formulae developed in medical research, both C5.0 decision tree and the baseline logistic regression model measured the morphological and syntactic complexity of health texts. Medical readability formulae focus on the average number of letters or syllables within words, or the average number of words within sentences, while machine learning models measure morphological and syntactic complexity using natural language features. Furthermore, machine learning models increase the dimensions of the analysis of linguistic accessibility of health texts.

The 2 new dimensions exploited by machine learning were information load and textual coherence. Information load was measured by natural language features including normalized frequency of noun phrases, density of content words, and TTR. Textual coherence and logical organization were measured by the 2 large categories of functional words, conjunctions (positive, negative) and pronouns (including personal pronouns). These quantitative models can serve as highly accurate, automated analytical tools to help predict linguistic accessibility of health translations in Chinese and represent a methodological advance from existing qualitative approaches in terms of the reliability, efficiency, and cost-effectiveness of the evaluation. The development of machine learning algorithms significantly improves upon the accuracy and reliability of current approaches to the evaluation of the linguistic accessibility of Chinese health information, especially Chinese health translations in relation to original health resources. Although the new algorithms developed were based on Chinese health resources, they can be adapted for other languages to advance current research in accessible health translation, communication, and promotion.

Automated predictive analyses of the linguistic accessibility of new health translations before their release to the public can significantly improve the cost-effectiveness, efficiency of bilingual and multilingual health education programs, and the use of health translation resources by the public. We developed new machine learning algorithms to help predict linguistic accessibility of health translations in Chinese, which represents a methodological advance from existing qualitative approaches in terms of the reliability, efficiency, and cost-effectiveness of the evaluation.

## Appendix

Multimedia Appendix 1 Mann-Whitney test of original and translated Chinese health information.

## Abbreviations

CCDC: China Center for Disease Control and Prevention
TTR: type-token ratio
WHO: World Health Organization
XGBoost tree: extreme gradient boosting tree
