# Supplementary material for: Predicting the Linguistic Accessibility of Chinese Health Translations: Machine Learning Algorithm Development
Source: JMIR Med Inform. 2021 Oct 7;9(10):e30588. doi: 10.2196/30588 (PMC8532010; doi:10.2196/30588)
Supplement: Multimedia Appendix 1 [file medinform_v9i10e30588_app1.docx]

Appendix 1 Mann Whitney U Test. of original and translated Chinese health information.

|  | **Variable** | **Original Resources** | | | **Translations** | | | **p-value** |
| --- | --- | --- | --- | --- | --- | --- | --- | --- |
|  |  | Mean | Min | Max | Mean | Min | Max |  |
| 1 | Average sentences per paragraph | 6.00 | 1.60 | 17.33 | 3.32 | 1.00 | 8.86 | 0.00 |
| 2 | TTR | 0.59 | 0.34 | 0.78 | 0.62 | 0.37 | 0.81 | 0.00 |
| 3 | Difficult words | 115.89 | 27.00 | 495.00 | 70.99 | 16.00 | 349.00 | 0.00 |
| 4 | Low-Stroke Characters | 407.76 | 105.00 | 1813.00 | 285.06 | 88.00 | 1241.00 | 0.00 |
| 5 | Middle-Stroke Characters | 83.16 | 18.00 | 379.00 | 51.90 | 14.00 | 258.00 | 0.00 |
| 6 | High-Stroke Characters | 0.74 | 0.00 | 11.00 | 0.45 | 0.00 | 17.00 | 0.01 |
| 7 | Average Strokes per character | 7.86 | 6.70 | 8.70 | 7.71 | 6.51 | 8.71 | 0.00 |
| 8 | 2-character words | 160.73 | 34.00 | 811.00 | 114.49 | 28.00 | 465.00 | 0.00 |
| 9 | 3-character words | 13.77 | 1.00 | 61.00 | 8.29 | 0.00 | 64.00 | 0.00 |
| 10 | average words per sentences | 10.83 | 7.61 | 20.27 | 11.90 | 6.32 | 21.50 | 0.00 |
| 11 | Simple sentences | 0.32 | 0.00 | 0.88 | 0.46 | 0.00 | 1.00 | 0.00 |
| 12 | ratio of noun phrases | 0.38 | 0.00 | 0.84 | 0.41 | 0.00 | 1.27 | 0.08 |
| 13 | frequency of noun phrases | 321.89 | 220.28 | 426.62 | 314.58 | 205.59 | 448.41 | 0.04 |
| 14 | content words | 245.27 | 74.00 | 1033.00 | 163.31 | 52.00 | 723.00 | 0.00 |
| 15 | Adverbs of Negation | 1.44 | 0.00 | 9.00 | 0.94 | 0.00 | 11.00 | 0.04 |
| 16 | Sentences with complex semantic categories | 14.42 | 1.00 | 70.00 | 7.61 | 0.00 | 35.00 | 0.00 |
| 17 | Density of content words | 0.83 | 0.75 | 0.92 | 0.81 | 0.69 | 0.90 | 0.00 |
| 18 | Pronouns | 2.36 | 0.00 | 18.00 | 1.47 | 0.00 | 11.00 | 0.01 |
| 19 | personal pronouns | 1.12 | 0.00 | 15.00 | 0.70 | 0.00 | 11.00 | 0.04 |
| 20 | Conjunctions | 14.00 | 2.00 | 47.00 | 11.53 | 2.00 | 45.00 | 0.01 |
| 21 | Positive conjunctions | 9.04 | 0.00 | 26.00 | 9.01 | 1.00 | 36.00 | 0.98 |
| 22 | Negative conjunctions | 2.03 | 0.00 | 16.00 | 1.44 | 0.00 | 8.00 | 0.03 |

*p*-values are derived from Mann Whitney U; a *p* value is considered significant if smaller than 0.05.
